# The electrophysiology correlation of the cognitive bias in anxiety under uncertainty

**DOI:** 10.1038/s41598-020-68427-y

**Published:** 2020-07-09

**Authors:** Shiyan Yang, Meng Zhang, Junye Xu, Li Wang, Zhaoxian Li, Feng Zou, Xin Wu, Yufeng Wang

**Affiliations:** Department of Psychology, Xinxiang Medical University, Henan, 453003 China

**Keywords:** Cognitive control, Consciousness

## Abstract

The intolerance of uncertainty (IU) model holds that excessive emotional response under uncertain conditions is conducive to the maintenance of anxiety, and individuals with a high anxiety level may exhibit a negative bias and experience anxiety when processing uncertain information. However, the dynamic electrophysiological correlation of this negative bias is not clear. Therefore, we used an adapted study–test paradigm to explore the changes in the electroencephalography (EEG) of subjects when processing uncertain cues and certain cues (certain neutral and certain threatening) and correlated the differences with anxiety level. The behavioral results showed that there was a significant positive correlation between the trait anxiety score and β value under the threatening condition, which indicated that individuals with high trait anxiety take a more conservative approach in the face of negative stimuli. The results of EEG showed that during the test stage, the components N1 and P2, which are related to early perception, had significant conditional main effects. Meanwhile, under uncertain conditions, the N1 peak was positively correlated with the state anxiety score. In the study stage, we found that the N400 component was significantly larger in the early study stage than in the late study stage under uncertain conditions. In sum, individuals with high anxiety levels had a negative bias in the early cue processing of the test stage, and anxiety did not affect the study stage.

## Introduction

A large number of studies have shown that patients with anxiety disorders have a wide range of cognitive impairments, including deficits in attention, executive functioning, learning and memory, and other abilities^1^. Patients with anxiety disorders have characteristic cognitive patterns, such as negative self-evaluation and negative thinking, and these factors are closely related to anxiety and avoidance behavior in daily life^2^. The core symptoms of anxiety are emotional, cognitive, and behavioral changes caused by negative expectations regarding uncertain events in the future^3^. In this process, excessive fear or worry will affect physical and mental health and cause inconvenience to daily life. The Attention Control Theory developed from Processing Efficiency Theory which was proposed by Eysenck and Calvo^4^ assumes that anxiety can impair the executive function of the goal-oriented attention system. In addition to reducing attentional control, anxiety increases attention to threat-related stimuli^5^. MacLeod^6^ used dot-probe task to compare the responses of clinically anxious patients and normal subjects to threat-related stimuli. The results showed that anxious subjects continued to turn their attention to threatening words, while the normal control group tended to distract themselves from these materials. A recent review summarized years of observations that lead to the same idea, which is that attention bias toward threats leads to the development of anxiety disorders^7^.

Spielberg proposed a classification method dividing anxiety into trait anxiety and state anxiety according to the duration of the anxiety^8^. State anxiety refers to anxiety with a short duration and a certain intensity of physiological response, which is a subjective feeling caused by tension and other factors. This feeling is controlled by the autonomic nervous system and can usually be perceived^9^. Individuals with high trait anxiety have cognitive biases that lead them to exaggerate the threat of external information or stimuli^10^. Due to the high consistency of behavior, brain structure, and functional connections between individuals with high trait anxiety and those with anxiety disorders, individuals with high trait anxiety are considered susceptible to anxiety disorders^11,12^.

Grupe^13^ defined anxiety as an expected emotional, cognitive, and behavioral change in response to the uncertainty of potential threats in the future. Previous studies have also confirmed that people with anxiety generally experience anxiety in the processing of uncertain relationships^14^. In the state of anxiety, people show excessive uneasiness and worry about uncertain events, attention bias (early attention bias), and persistent attention (continuous processing of negative information)^6,15^. Reasoning/judgment under uncertainty refers to the cognitive process in which individuals engage in reasoning and decision-making based on known uncertain information or incomplete information, combined with their own cognitive schema and knowledge experience, which has an impact on future behavior^16^. One of the main theories of Generalized Anxiety Disorder (GAD) holds that intolerance of uncertainty (IU) plays the central role of a cognitive bias that interferes with information processing (including decision-making)^17^. According to the IU model, in the case of uncertainty, excessive emotional response is conducive to the development and maintenance of anxiety^18^. A study in a large sample of 1,092 young people also showed that IU has a significant moderating effect on the relationship between daily stress and worry^19^. Furthermore, the results of Reuman’s experiments showed that when people face greater threats, when they are clearly uncertain, it can lead to higher levels of anxiety and safety-seeking behavior or evasive behavior^20^. In people’s daily life, reasoning and decision-making based on uncertain information are the most common form of cognition^21^. Although previous studies have shown a significant correlation between uncertainty and anxiety, most of the studies on uncertainty have been about the handling of events after the emergence of uncertain cues, such as probability-based gambling and reward evaluation^22–24^, and did not adequately explain what kinds of rules people follow in uncertain situations or the processing mechanism of uncertain information.

In order to clarify the dynamic trajectory of processing uncertain cues in individuals with high anxiety levels, we used an adapted study–test paradigm combined with an event-related potential (ERP) methodology to collect experimental electroencephalography (EEG) data for analysis. We add a cued anticipation task to the study–test paradigm. The study–test paradigm^25^ is a classical memory paradigm. The cued anticipation task^26^ is a task in which cues are displayed before presenting pictures, with different cues corresponding to different conditions. The purpose of the cued anticipation task was to explore the changes in brain regions during preconditioning when subjects processed cues^26^. The Williams^27^ Study compared children with and without anxiety on a cued anticipation task involving certain and uncertain cues. One of the certain cues always preceded the presentation of a neutral faces. The other certain cue always preceded the display of fearful faces. The uncertain cue was equally likely to be preceded by a neutral or fearful face. The researchers found that children with anxiety showed greater amygdala activation to uncertain cues compared to children without anxiety symptoms. Additionally, amongst the children with anxiety disorders, faces preceding the uncertain cue elicited greater amygdala activation than the faces preceded the certain cues. According to previous studies^28^, the visual cortex, bilateral fusiform gyrus and right parahippocampal gyrus are significantly active when processing the uncertain cues, which is similar to the neural mechanism of processing threatening cues, so we speculated that under uncertain conditions, the processing of uncertain information may be similar to the processing of certain threatening information, and the differential ERP may be a component related to early perceptual processing. In this experiment, we chose three irregular graphics as cues, corresponding to a certain neutral condition (neutral pictures), certain threatening condition (threatening affective pictures), and uncertain condition (neutral/threatening affective pictures), and analyzed the processing of cues in the study stage and the test stage. An ERP study using the Wheel of Fortune gambling task found that when people process uncertain cues, they treat them as negative signals, which triggered a larger P200 amplitude^29^. Therefore, we hypothesized that the P200 amplitude would be similar to processing certain threatening cues when processing uncertain cues. We also hypothesized that during the encoding of cues by subjects in the test stage, there would also be differences in the early components of perception between the uncertain and certain condition, such as the early visual perceptual N1 ERP component changes^30^. In support of this idea, a study found that the N1 amplitude changes when participants use numbers compared to verbal probabilities to represent the uncertainties that they face on a decision-making task. Specifically, the N1 amplitude elicited by the verbal probability was greater than that elicited by the numerical probability at the Oz electrode, but the opposite pattern was observed at the Fz electrode^31^. An ERP study of attention found that increased attention triggered a smaller N1 amplitude^32^. Hence, we hypothesized that the N1 amplitude would get smaller and that this change would be related to the anxiety level. The earliest study of the N400 component found that it can be triggered by semantically inappropriate words, such as “I take coffee with cream and *socks*”^33^*.* Later, Kutas marshalled three decades of research on the N400, linking it to almost every aspect of language processing and highlighting its role in exploring semantic memory^34^. Thus, we speculated that the learning of uncertain cues might elicit the N400 component, particularly during the study stage, given its role in semantic learning^35,36^.

## Results

### Behavioral results

**Figure 1:**
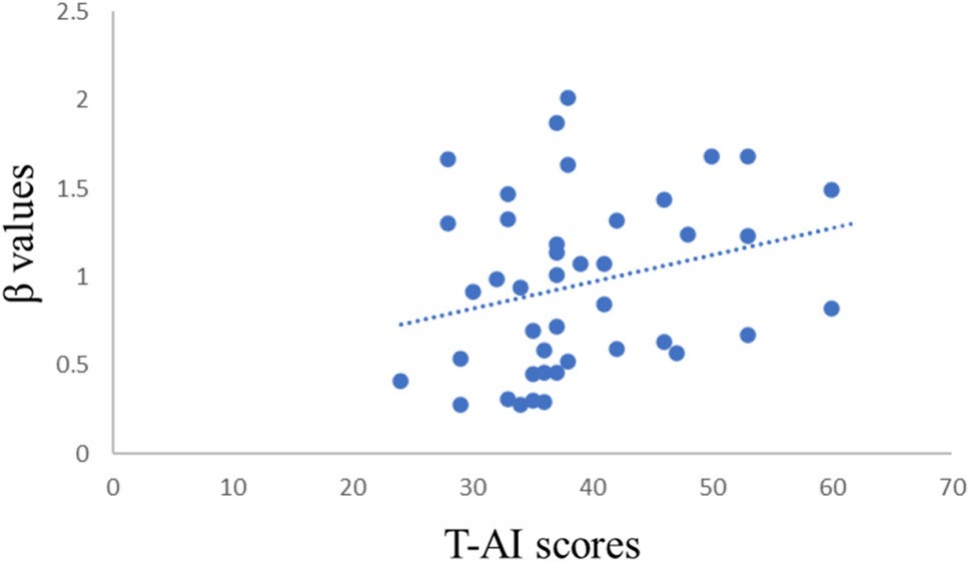
A scatter plot of the relationship between trait anxiety and β under the certain threatening condition (r = 0.253).

We found a main effect of the correct rate in recognition for cue type (*F*(2, 123) = 41.65, *p* < 0.001, *η*_*p*_^2^ = 0.404), the follow-up comparisons demonstrated that this was driven by certain neutral condition, which had lower correct rate compared to the certain threatening condition (*p* < 0.001) and uncertain condition (*p* < 0.001). In addition, uncertain condition and certain threatening condition did not differ in the correct rate (*p* = 0.59). The analysis of *d*′ showed a main effect of condition (*F*(2, 123) = 21.27, *p* < 0.001, *η*_*p*_^2^ = 0.257), follow-up comparisons demonstrated that this was driven by certain neutral condition, which had lower correct rate compared to the certain threatening condition (*p* < 0.001) and uncertain condition (*p* < 0.001). Furthermore, uncertain condition and certain threatening condition did not differ in the correct rate (*p* = 0.43). At the same time, we also found the conditional main effect of *β* (*F*(2, 123) = 27.00, *p* < 0.001, *η*_*p*_^2^ = 0.305), the follow-up comparisons showed that the certain neutral condition had higher *β* compared to the uncertain condition (*p* < 0.001), and the uncertain condition had higher *β* compared to the certain threatening condition (*p* = 0.031).The *β* value was positively correlated with the trait anxiety score in the certain threatening condition (*r* (42) = 0.253, *p*_uncorrected_ < 0.05). (see Table 1 and Fig. 1).

**Table 1:** Means and SDs of ACC, *d*′ and β for different conditions.

Type	ACC (%)	*d*′	β
Certain threatening	81.52 ± 8.17	1.99 ± 0.70	0.96 ± 0.49
Certain neutral	67.63 ± 6.48	1.18 ± 0.49	2.52 ± 1.38
Uncertain	79.39 ± 7.80	1.80 ± 0.58	1.51 ± 0.87

### ERP results

#### Test stage

The results of repeated measurements of N1 (100–160 ms) peak in the test stage showed that an interaction between regions and conditions was significant (*F*(12, 35) = 3.064, *p* < 0.005, *η*_*p*_^2^ = 0.512), subsequent simple effect analysis demonstrated that the N1 peak amplitude differed between three conditions over the FP, F, FC, and PO regions. The results of repeated measurements of N1 (100–160 ms) latency in the test stage showed that an interaction between regions and conditions was significant (*F*(12, 35) = 2.353, *p* = 0.024, *η*_*p*_^2^ = 0.447), subsequent simple effect analysis demonstrated that the N1 latency differed between three conditions over the P and PO regions.

**Figure 2:**
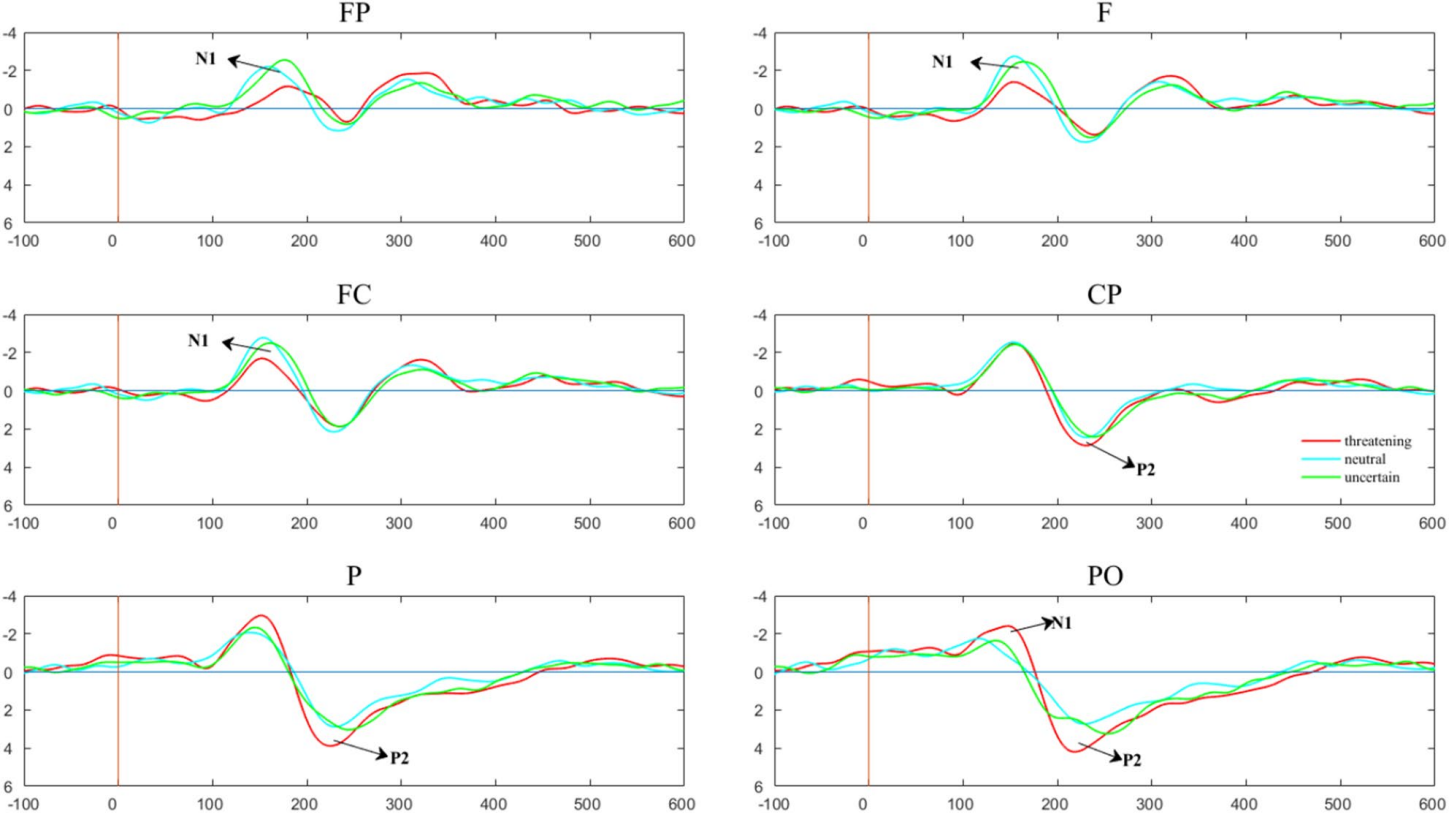
The waveform figure over the FP (prefrontal) region, the F (frontal) region, the FC (frontal central), the centroparietal (CP) region, the parietal (P) region and the posterior occipital (PO) region.

**Figure 3:**
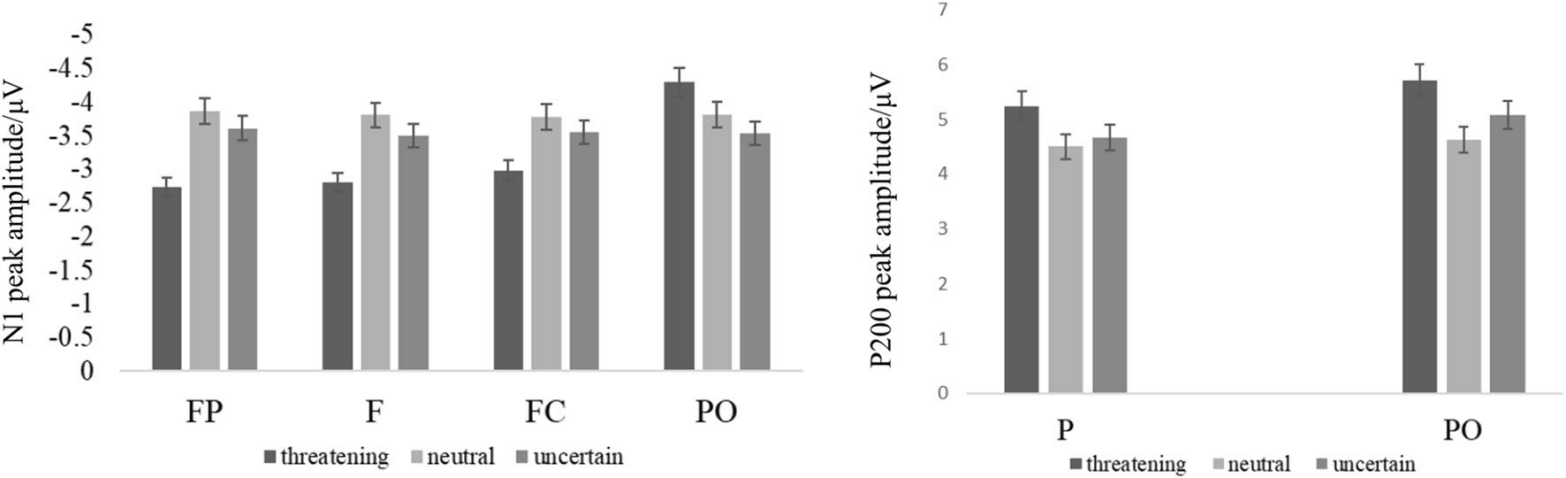
The amplitude difference Bar Diagram of N1 (100–160 ms) peak amplitudes in FP region, F region, FC region, PO region and P2 (200–300 ms) peak amplitudes in P region and PO region during the test stage.

Meanwhile, the results of repeated measurements of P2 (200–300 ms) peak in the test stage showed that an interaction between regions and conditions was significant (*F*(12, 35) = 3.32, *p* < 0.005, *η*_*p*_^2^ = 0.532), subsequent simple effect analysis demonstrated that the P2 peak amplitude differed between three conditions over the P, and PO regions. The results of repeated measurements of P2 (200–300 ms) latency in the test stage showed that an interaction between regions and conditions was significant (*F*(12, 35) = 2.134, *p* = 0.014, *η*_*p*_^2^ = 0.297), subsequent simple effect analysis demonstrated that the P2 latency differed between three conditions over the CP, P, and PO regions. For detailed data, please see Table 2. The amplitudes of N1 and P2 are shown in Figs. 2 and 3.

**Table 2:** The results of repeated measurements of N1 (100–160 ms) and P2 (200–300 ms) peak and latency in the test stage (corrected by Bonferroni).

N1 (100–160 ms)					
	df	F	*p* value	η_p_^2^	Post-hoc
N1 peak					
FP	2,45	5.558	0.007	0.198	Threatening > neutral *p* = 0.011
F	2,45	5.848	0.006	0.206	Threatening > neutral *p* = 0.009
FC	2,45	4.722	0.014	0.173	Threatening > neutral *p* = 0.021
PO	2,45	4.28	0.02	0.16	Threatening < uncertain *p* = 0.022
N1 latency					
P	2,45	3.979	0.026	0.15	Threatening > neutral *p* = 0.023
PO	2,45	5.971	0.005	0.21	Threatening > neutral *p* = 0.003 Threatening > uncertain *p* = 0.028

P2 (200–300 ms)					
	df	F	*p* value	η_p_^2^	Post-hoc
P2 peak					
P	2,45	4.735	0.014	0.174	Threatening > neutral *p* = 0.011
PO	2,45	9.862	0.000	0.305	Threatening > neutral *p* < 0.000
P2 latency					
CP	2,45	5.633	0.007	0.2	Threatening < neutral *p* = 0.048 Threatening < uncertain *p* = 0.009
P	2,45	6.964	0.002	0.236	Threatening < uncertain *p* = 0.002
PO	2,45	4.918	0.012	0.179	Threatening < neutral *p* = 0.027 Threatening < uncertain *p* = 0.021

We analyzed the correlations between the subject’s anxiety level and the peak and latency of the ERP component N1 and P2. The results showed that the P2 peak was negatively correlated with the trait anxiety score under the uncertain condition over the P region (*r* (45) =  − 0.252, *p*_uncorrected_ = 0.041) and there was no significant correlation between P2 latency and trait anxiety scores under the uncertain condition over the P region(*r* (45) = 0.128, *p*_uncorrected_ = 0.196). Then we analyzed its correlation with the state anxiety score and found that the N1 peak was positively correlated with the state anxiety score under uncertain conditions over the FC region (*r* (45) = 0.333, *p*_corrected_ = 0.044)); meanwhile, P2 latency was negatively correlated with the state anxiety score under uncertain conditions over the F region (*r* (45) =  − 0.357, *p*_corrected_ = 0.028) and there was no significant correlation between P2 peak and state anxiety scores under the uncertain condition over the F region(*r* (45) = 0.109, *p*_uncorrected_ = 0.232)..

Finally, we calculated the difference between the certain threatening condition and uncertain condition and conducted a correlation analysis between the difference and the level of anxiety. The difference in the N1 peak was negatively correlated with the state anxiety score of the subjects in the PO region (*r* (45) =  − 0.313, *p*_uncorrected_ = 0.016) and the P region (*r* (45) =  − 0.275, *p*_uncorrected_ = 0.031); the difference in the N1 latency was negatively correlated with the trait anxiety score of the subjects in the FP region (*r* (45) =  − 0.314, *p*_uncorrected_ = 0.016). The results were corrected by the Benjamini–Hochberg procedure.

#### Study stage

During the test stage, we found that the subjects had a negative bias in processing uncertain cues based on the behavioral results for *d*′, and the ERP results showed that under uncertain condition, the difference in the N1 peak of the test stage was negatively correlated with the state anxiety score of the subjects. we speculated that the coding bias might be caused by the influence of anxiety in the study stage, so we divided the study stage into the early study stage and the late study stage from the halfway point for further analysis.

**Figure 4:**
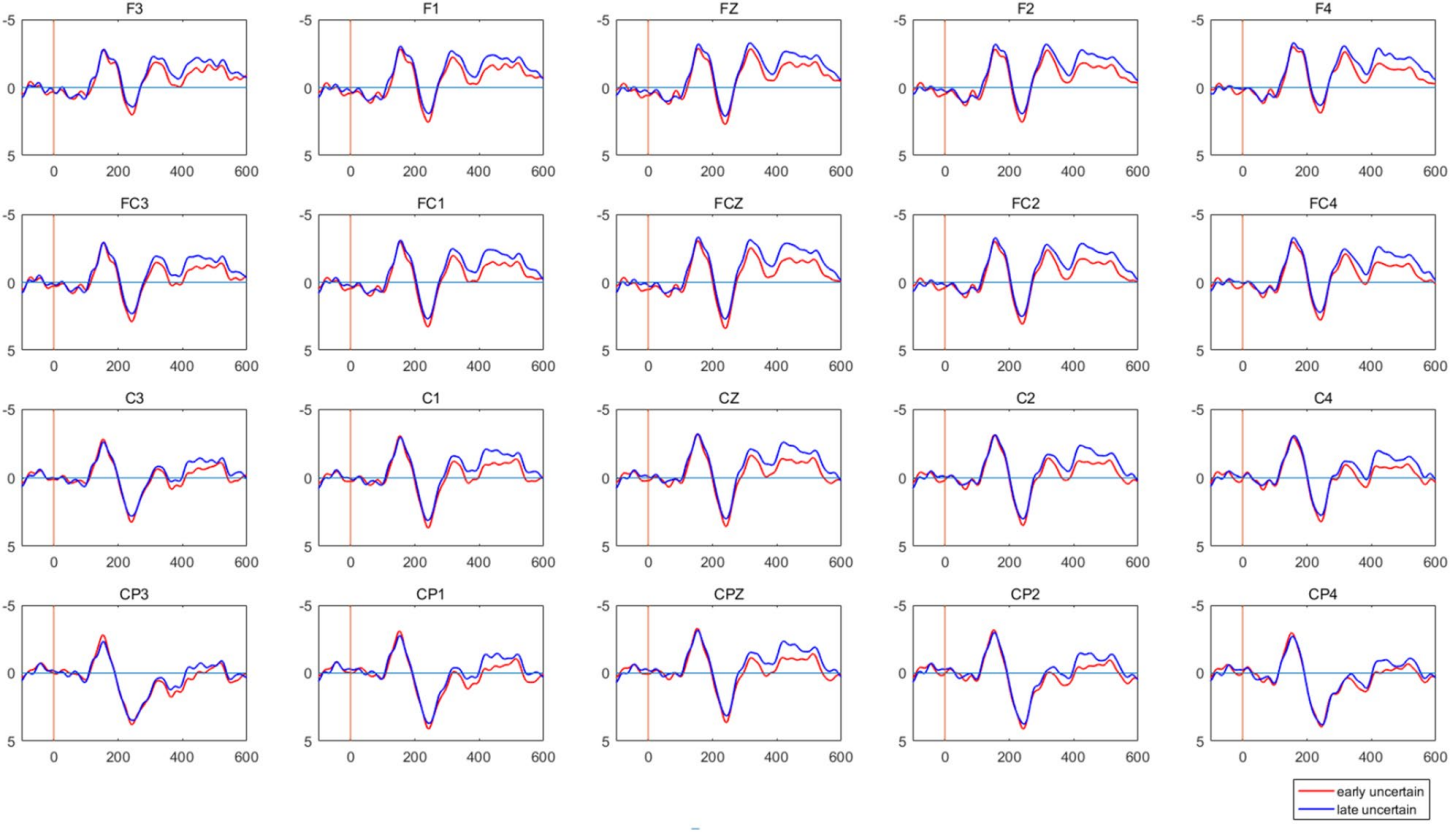
When learning uncertain cues, the difference of N400 (410–460 ms) between the early study stage and the late study stage.

The repeated measures ANOVA of the average amplitude of N400 (410–460 ms) showed that the main effects of region [*F*(3, 44) = 23.107, *p* < 0.000, *η*_*p*_^2^ = 0.612], time [*F*(1, 46) = 6.815, *p* = 0.012, *η*_*p*_^2^ = 0.129], and the interaction between region and type [*F*(6, 41) = 4.187, *p* = 0.002, *η*_*p*_^2^ = 0.380] were significant. Meanwhile, simple main effects analysis showed that the EEG activation was significantly greater under the certain neutral condition than under the uncertain condition over the central (C) region [*F*(2, 45) = 4.065, *p* = 0.024, *η*_*p*_^2^ = 0.153] and the centroparietal (CP) region [*F*(2, 45) = 5.265, *p* = 0.009, *η*_*p*_^2^ = 0.190]. The interaction between time and type was significant, *F*(2, 45) = 4.187, *p* = 0.032, *η*_*p*_^2^ = 0.142. Furthermore, in the late study stage, the EEG activation was significantly higher under the certain neutral condition compared to the uncertain condition, *F*(2, 45) = 5.113, *p* = 0.010, *η*_*p*_^2^ = 0.185; as for the uncertain condition, the EEG activation was significantly higher in the late study stage than in the early study stage, *F*(1, 46) = 8.373, *p* = 0.006, *η*_*p*_^2^ = 0.154 (Fig. 4).

Finally, we calculated the difference among the three conditions in the early study stage and the later study stage, and then we calculated the difference between the uncertain condition and the certain neutral condition or certain threatening condition and conducted a correlation analysis between the difference and the level of anxiety; the results showed that there was no significant correlation (*p* > 0.05, *n* = 47).

## Discussion

We are faced with all kinds of uncertain events with incomplete information in our lives, so individuals are often in the state of processing uncertain information. Previous studies have found that individuals with high trait anxiety show negative bias and anxiety when dealing with uncertain information^37^. In order to clarify the dynamic electrophysiological mechanism of this negative bias, we used the adapted study–test paradigm, which represented the certain negative conditions, certain neutral conditions, and uncertain conditions by setting three kinds of graphical cues, exploring the EEG activity in the process of uncertain cue coding and its relationship with anxiety level.

The behavioral data showed that the ACC of the neutral condition was the lowest, and there was no significant difference between the threatening condition and the uncertain condition. In signal detection theory, *d*′ reflects subjects’ objective susceptibility, and *β* reflects their reaction bias to stimuli^38^. The larger *d*′ for the certain threatening condition and uncertain condition compared with the certain neutral condition showed that the subjects exhibited a certain threatening processing deviation under uncertain conditions and tended to perceive the uncertain conditions as threatening conditions. The *β* value was positively correlated with the trait anxiety score in the certain threatening conditions, indicating that individuals with high trait anxiety had higher *β* values; the higher the *β* values, the more stringent the criteria are, so individuals with high trait anxiety take a more conservative approach in the face of negative stimuli.

### Test stage

Next, we analyzed the EEG data from the test stage, with N1 reflecting the earliest cognitive processing stage of this study. Previous studies have shown that N1 is related to the allocation of attention, and people tend to allocate more attention to negative stimuli^39–41^. In this study, the N1 peak caused by threatening cues was the largest over the FP, F, and FC regions, indicating that the activation degree of threatening cues was the smallest in the early cognitive processing of individuals, which was consistent with the results of previous studies^32^. The N1 latency represents the early or late start of cognitive processing or the speed of processing, the shorter the latency period, the higher the degree of automated processing^42^, in our study, the N1 latency caused by threatening cues was the largest over the P and PO regions, indicating that the degree of automatic processing of threatening cue is lower. Furthermore, our results showed that the N1 peak under the uncertain condition was positively correlated with the state anxiety score over the F region. The higher the anxiety score, the smaller the N1 amplitude, which indicated that anxious individuals were more inclined to perceive uncertain cues as threatening cues. Our correlation analysis of the difference between the uncertain condition and the certain threatening condition with anxiety scores also found that the higher the anxiety score, the smaller the difference between the two conditions, exhibiting the same results for both peak and latency periods, further demonstrating that high-anxiety individuals tend to process uncertain cue perception as a threatening cue.

In previous studies, P2 components were found to be affected by attention, memory, hearing, vision, and other factors^43–45^. However, most studies on P2 have focused on visual processing, and P200 is considered to be involved in the encoding and matching of early visual features^46^. In our study, the results showed that the peaks of P2 were higher in the certain threatening condition over the P and PO regions, the latency of P2 were earlier in the certain threatening condition over CP, P and PO regions. P2 latency was negatively correlated with the state anxiety score under the uncertain condition over the F region. However, the peak of P2 had no relationship with the anxiety scores. Therefore, our results confirm that the changes of P2 is only due to the difference caused by the attention processing of certain cues and uncertain cues^29^, and people will be quicker to notice threatening cues and showed increased attention to certain threatening cues, but individuals with high anxiety may notice the stimulus more quickly. In other words, in daily life, both highly anxious individuals or ordinary individuals have a negative deviation, that is, increased the attention to threatening stimuli.

### Study stage

Furthermore, for the study stage, the EEG data were divided into the early study stage and the late study stage at the halfway point. Because in the middle of the break, the subjects were informed of the connection between the cues and affective pictures and understood the rules, the early study stage represents the uncertainty of learning, and the late study stage represents the knowledge of the meaning of uncertain cues. N400 is generally caused by unexpected stimuli, and its amplitude is affected not only by the expected value but also by the characteristics of the stimulus itself. For example, the amplitude of N400 is higher in response to low-frequency words than in response to high-frequency words and higher in response to unfamiliar faces than in response to familiar faces^47^. After collating three decades’ worth of research on N400, Kutas concluded that N400 is associated with almost all aspects of language processing and emphasized the role of N400 in exploring semantic memory^34^. Therefore, the differences in N400 between the early study stage and the late study stage in this study can be understood as the part of semantic memory processing activated by the participants when processing uncertain cues. Finally, we calculated the difference among the three conditions before and after the study, referring to the uncertain condition difference as UN, the certain neutral condition difference as CN, and the certain threatening condition difference as CF. Then we conducted a correlation analysis between UN-CN and UN-CF and anxiety scores. The results indicated that there was no difference in learning of uncertain cues between highly anxious individuals and normal individuals, and the study stage may not be the cause of the negative bias of individuals with high anxiety levels.

## Conclusion

In general, the behavioral results of our experiments indicated a correlation between *β* and trait anxiety. Meanwhile, the ERP results showed changes in components N1 and P2 in two early stages, first for the N1 component, with the N1 peak having a negative correlation with the state anxiety score under the uncertain condition; followed by the P2 component, in which there was a negative early processing deviation, but this deviation had nothing to do with uncertainty. Two conclusions can be drawn from our findings. First, regardless of the level of anxiety, individuals tend to adopt conservative strategies and pay more attention to negative stimuli, mainly reflected in β and P2. Second, we found an earlier component N1, whose peak was positively correlated with the state anxiety score under the uncertain condition. This further verified that uncertain situations may be an important inducer of anxiety susceptibility for highly anxious individuals^48^. Moreover, our study showed that the influence of uncertain situations on anxious individuals mainly occurred in a very early stage, suggesting that we should pay more attention to the sensory coding and sensory gating of anxious individuals in the face of uncertain situations in the future.

In our results, some components are only related to trait anxiety, but not to state anxiety, while some components are related to state anxiety but not to trait anxiety. We think that this may be due to the different measurement targets of the STAI. The trait anxiety inventory(TAI) is used to evaluate people's frequent emotional experience^49,50^, measuring a stable personality trait and testing a tendency of anxiety, while the State anxiety inventory(SAI) is used to assess fear, tension, anxiety and neurotic experience or feelings of immediate or recent specific time or situation, and is to detect a state of anxiety^49,51^. Therefore, it may exist that some components are more sensitive to state anxiety and some components are more sensitive to trait anxiety in the process of uncertain processing. This also provides a way for us to study anxiety more deeply in the future.

## Methods

### Participants

We recruited 51 healthy undergraduates from Xinxiang Medical University via an advertisement and paid them 15 RMB for their participation. Four participants were excluded, two of whom failed to complete the experiment, while the other two showed excessive artifacts in the EEG signals (which with 7% of the total trials were rejected), so their data were rejected from the statistical analysis. Therefore, 47 participants (24 females and 23 males, mean age = 20.55 years old) were included in the final analysis. All participants were right-handed, with normal or corrected-to-normal vision, and had no history of neurological or mental disorders. All participants volunteered to sign informed consent forms, and the institutional ethics committee of Xinxiang Medical University approved the study protocol. The experimental methods were carried out in accordance with the approved guidelines.

### Materials

The experiment consisted of the presentation of two kinds of pictures, one being the cue picture and the other being the affective picture. There were three types of cue pictures—triangle, circle, and square—and the affective pictures were taken from the International Affective Picture System (IAPS)^52^, with a total of 216 pictures (108 neutral pictures and 108 threatening pictures) used in the experiment. We used 108 pictures (54 neutral pictures and 54 threatening pictures) in the study stage and 216 pictures in the test stage. Therefore, the 108 pictures (54 neutral pictures and 54 threatening pictures) presented in the study stage appeared twice in the entire process of the experiment.

The State-Trait Anxiety Inventory (STAI)^53^ is a self-rating scale consisting of 40 items; the first 20 questions constitute the state anxiety scale, which reflects the experience of feeling of fear, tension, and nervousness at a particular time, either currently or recently, and the last 20 questions constitute the trait anxiety scale, used to assess the individual’s frequent emotional experiences. The items are rated on a 4-point scale (1 = seldom/never, 4 = always/very often). And the experimental program was presented using E-Prime1.0 software^54^.

### Procedure

First, 108 neutral pictures and 108 threatening pictures were selected from the IAPS^52^ as experimental materials. Using the adapted study–test paradigm, the whole experiment was divided into two stages. Before the beginning of the experiment, the subjects filled in their general personal data, and then we used the STAI to assess the anxiety level of the subjects. The subjects were asked to sit on a comfortable stool 80 cm from the screen and react to the stimulus on the screen according to the instructions. All of the stimuli were presented against a black background. The behavioral data and EEG data of the subjects were recorded throughout the experiment.

**Figure 5:**
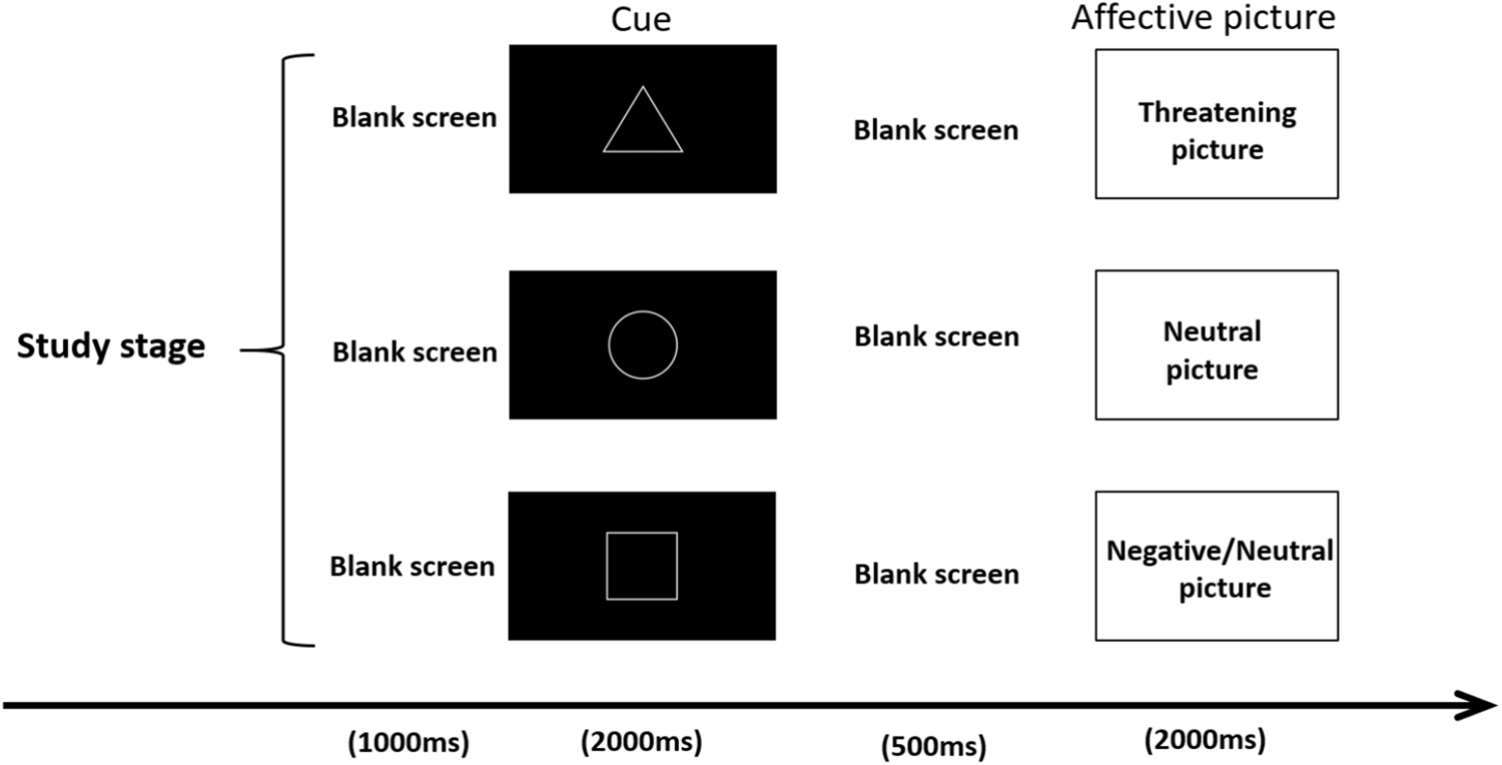
The study stages. Each trial started with a blank screen for 1,000 ms; then the cue picture (triangle, circle or square) appeared in the center of the screen for 2,000 ms, followed by a blank screen for 500 ms; after that, the affective picture was presented for 2,000 ms.

The experiment was divided into two parts. The first part was the study stage (see Fig. 5), in which the subjects learned the relationship between the cue picture and the affective picture. The three kinds of cue pictures represented three corresponding relationships: the triangle corresponded to threatening pictures, the circle corresponded to neutral pictures, and the square corresponded to uncertain emotional conditions (the affective picture appeared after the square cue picture, with neutral pictures and threatening pictures each accounting for 50% of the trials, and the threatening and neutral pictures were presented at random). The study stage contained two blocks, and each block included 54 trials consisting of 27 neutral pictures and 27 threatening affective pictures, correspond to three cues, with 18 trials for each cue. Each trial started with a blank screen for 1,000 ms; then the cue picture (triangle, circle, or square) appeared in the center of the screen for 2,000 ms, followed by a blank screen for 500 ms; after that, the affective picture was presented for 2,000 ms. Subjects were instructed to take a short break between the two blocks; during the break, the subjects were told the corresponding relationships between the cue pictures and the affective pictures. After the study stage, the subjects completed a simple task, in which, after seeing a cue picture, they were required to predict the affective pictures’ emotional valence. When the subjects could accurately predict the affective pictures’ valence after the three kinds of cues, they could enter the test stage.

**Figure 6:**
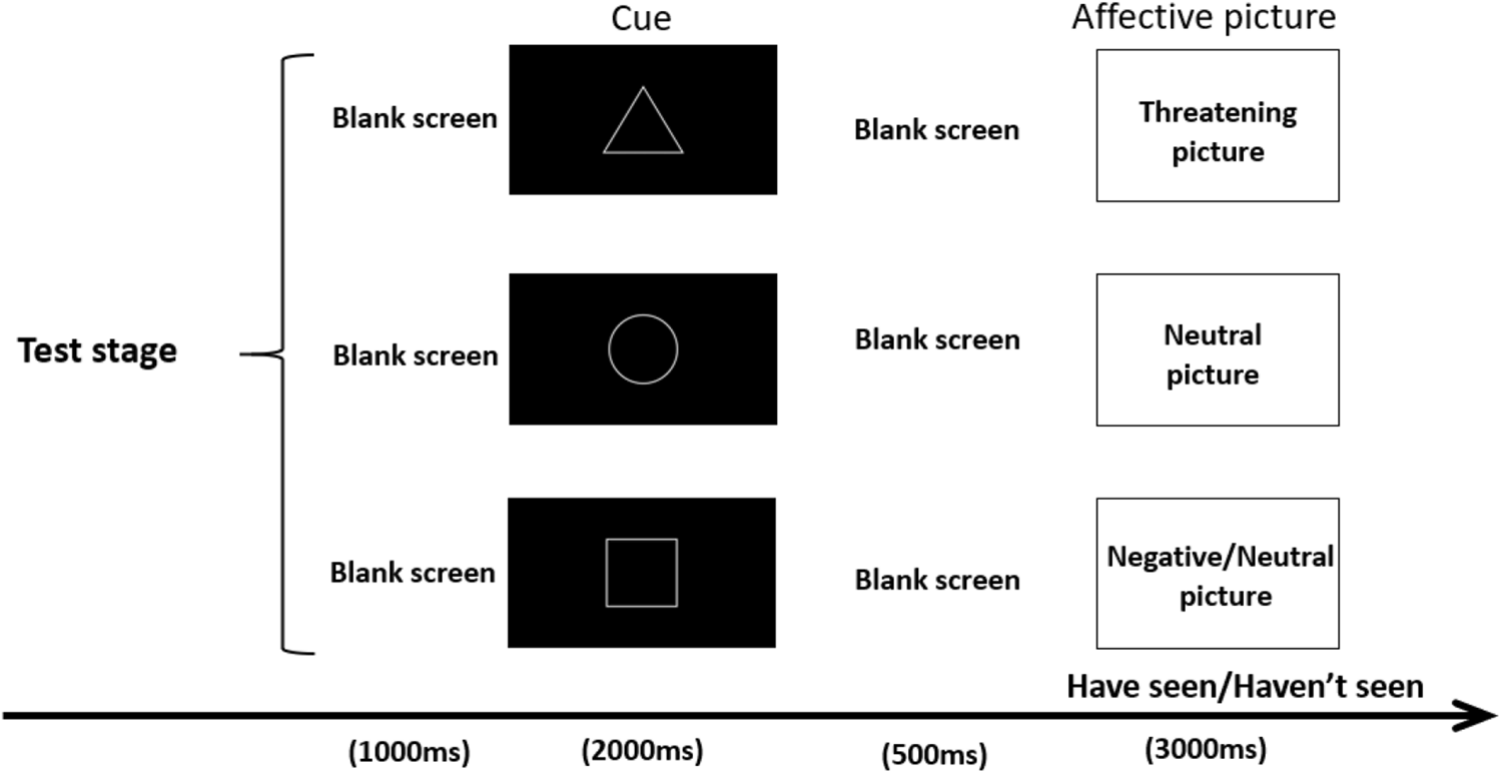
Test stage. Each trial started with a blank screen for 1,000 ms; then the cue picture (triangle, circle, or square) appeared in the center of the screen for 2,000 ms, followed by a blank screen for 500 ms; after that, the affective picture was presented for 3,000 ms (half of the affective pictures presented in the test stage appeared in the study stage). And the subjects needed to judge whether the presented pictures had appeared during the study stage. If they thought they had seen it in the study stage, they pressed “P”; if not, they pressed “Q”.

The test stage (see Fig. 6) consisted of 216 trials, again divided into two blocks. Each trial started with a blank screen for 1,000 ms; then the cue picture (triangle, circle, or square) appeared in the center of the screen for 2,000 ms, followed by a blank screen for 500 ms; after that, the affective picture was presented for 3,000 ms (half of the affective pictures presented in the test stage appeared in the study stage), and the subjects needed to judge whether the presented pictures had appeared during the study stage. If they thought they had seen the picture in the study stage, they pressed “P”; if not, they pressed “Q”. The trials were presented randomly in the test stage, and the break time between the two blocks was determined by the subject.

### ERP data acquisition

Brain electrical activity during the task was recorded from 64 Ag–AgCl scalp sites according to the international 10–20 system; EEG electrodes were referenced to Cz (the middle point between the two ears) and re-referenced offline to linked mastoids. Two pairs of electrodes were used to record the horizontal and vertical electrooculograms: one pair of electrodes was placed parallel to the upper and lower part of the left eye, and the other pair of electrodes was placed at 10 mm from the lateral canthi. The impedance was less than 5 kΩ for recording. The sampling rate was 500 Hz, and online band-pass filtering (0.1–100 Hz) was applied with a 50-Hz notch filter.

### Data analysis

#### Behavioral data

We analyzed the subjects’ accuracy in the test stage and indicators in signal detection theory (*d*′ and *β*); according to signal detection theory, if a picture of an object that was chosen from the study stage appeared in the test stage, and the participants made a correct judgment, it was marked as a HIT, whereas if the participants made a wrong judgment, it was marked as a MISS. If a picture of an object that was not chosen from the study stage appeared in the test stage, and the participants made a correct judgment, then it was marked as a CORRET REJECTION, whereas if the participants made a wrong judgment, then it was marked as a FALSE ALARM. Then, the P(H) and the P(FA) in the test stage were analyzed by the following formulas:

$$ \begin{gathered} {\text{P}}({\text{H}}) \, = {\text{ n}}({\text{HIT}})/[{\text{n}}({\text{HIT}}) \, + {\text{ n}}({\text{MISS}})], \hfill \\ {\text{P}}({\text{FA}}) \, = {\text{ n}}({\text{FALSE}}\;{\text{ ALARM}})/[n({\text{FALSE}}\;{\text{ ALARM}}) \, + \, n({\text{CORRET}}\;{\text{ REJECTION}})]. \hfill \\ \end{gathered} $$

The P(H) and the P(FA) in the test stage were translated to O(H) and O(FA) using PZO translation. Then, the likelihood ratio (*β*) in the test stage was analyzed by the following formulas:

$$ \begin{gathered} \beta = {\text{ O}}({\text{H}})/{\text{O}}({\text{FA}}). \hfill \\ {\text{d}}^{\prime} = {\text{ Z}}({\text{H}}) \, - {\text{ Z}}({\text{FA}}). \hfill \\ \end{gathered} $$

Higher *β* values (the likelihood ratio or decision criterion; the higher the β value, the stricter the criterion) indicate worse memory performance in this study. Higher *d*′ values (discrimination index; the higher the *d*′ value, the stronger the sensitivity) indicate better memory performance in this study. We performed one-way analysis of variance (ANOVA) with condition (certain threatening, certain neutral, and uncertain) as the within-subject factor. In addition, we calculated the differences in the data among the uncertain context, certain threatening context, and certain neutral context. All of the *p*-values were corrected using the Bonferroni adjustment. Then the correlations between these differences and the anxiety level of the subjects were analyzed.

#### ERP data analysis

All of the ERP data were analyzed using the MATLAB toolbox (R2016b, MathWorks, Inc.). We set the high-pass filter of the EEG signal to 0.05 Hz and the low-pass filter to 30 Hz; four channels (VEO, HEO, CB1, CB2) weren’t included in our artifact analysis, and then we performed independent component analysis (ICA) for eye movement correction, manually removing eye movement and eye drift components^55,56^ using EEGLAB software version 4.5^57^. In artifact analysis of ERP data, about 7% of the total trials were deleted due to excessive noise and bad segments by an automated script identifying artifacts based on ± 100 μV amplitude parameters.

We first analyzed the ERP data from the test stage. The epochs of 300 ms before the stimulus and 800 ms after the stimulus were extracted from continuous EEG, and the baseline was corrected according to the window of 300 ms before the stimulus onset. Trials with bad signals or incorrect responses were excluded from analysis. The analyzed positions including the FP (prefrontal) region (i.e., FP1, FPZ, FP2, AF3, AF4), the F (frontal) region (i.e., F5, F3, F1, FZ, F2, F4, F6), the FC (frontal central) region (i.e., FC5, FC3, FC1, FCZ, FC2, FC4, FC6), the C (central) region (i.e., C5, C3, C1, CZ, C2, C4, C6), the CP (central parietal) region (i.e., CP5, CP3, CP1, CPZ, CP2, CP4, CP6), the P (parietal) region (i.e., P5, P3, P1, PZ, P2, P4, P6), and the PO (posterior occipital) region (i.e., PO5, PO3, POZ, PO4, PO6). We analyzed the peak latencies of N1 (100–160 ms) and P200 (200–300 ms) using a 3 (cue type: neutral, threatening, uncertain) × 7 (region: FP, F, FC, C, CP, P, PO) repeated measures ANOVA. Then the correlation between ERP data and anxiety level was analyzed, a total of seven brain regions (FP, F, FC, C, CP, P, PO) were included in the ERP data analysis. Taking the correlation analysis of N1 peak as an example, we ran four related tests on each brain region, which were related to trait anxiety scores under uncertain conditions and state anxiety scores under uncertain conditions. the difference between the uncertain condition minus the certain threatening condition is related to the trait anxiety score, and the difference is related to the state anxiety score. FDR correction was performed in each brain region. Similarly, for N1 latency, P2 peak and P2 latency, we run the correlational tests and FDR corrections in the same process as in N1 peak.

For the study stage, the EEG data were divided into the early study stage and the late study stage at the halfway point. The epochs were selected at the same time as for the test stage. Based on the differential topographical representation between the early study stage and the late study stage, we selected 20 electrode positions for analysis, including the F (frontal) region (i.e., F3, F1, FZ, F2, F4), FC (frontal central) region (i.e., FC3, FC1, FCZ, FC2, FC4), C (central) region (i.e., C3, C1, CZ, C2, C4), and CP (central parietal) region (i.e., CP3, CP1, CPZ, CP2, CP4), and analyzed the mean amplitudes of N400 (410–460 ms) components. The EEG changes between the early study stage and late study stage were analyzed using a 2 (time: early, late) × 3 (cue type: neutral, threatening, uncertain) × 4 (region: F, FC, C, CP) repeated measures ANOVA. Then the correlation between ERP data and anxiety level was analyzed.

In general, the peak test is often used for early ERP components to see the peak and incubation period, while the average amplitude test is often used for late ERP components^58,59^. Therefore, for the test stage, we analyzed the peak and latency of the ERP components N1 and P2. For the study stage, we analyzed the average amplitude of the ERP component N400. All of the analysis were performed using SPSS Statistics software version 23.0 (IBM, Armonk, NY, USA). All of the ANOVA results were Bonferroni corrected, and the results of correlation analysis were corrected by the Benjamini–Hochberg procedure.
